# The Food Literacy Action Logic Model: A Tertiary Education Sector Innovative Strategy to Support the Charitable Food Sectors Need for Food Literacy Training

**DOI:** 10.3390/nu11040837

**Published:** 2019-04-12

**Authors:** Tanya Lawlis, Ros Sambell, Amanda Douglas-Watson, Sarah Belton, Amanda Devine

**Affiliations:** 1 Discipline Nutrition and Dietetics, University of Canberra, Canberra, ACT 2601, Australia; sarah.belton@live.com.au; 2 School of Medical and Health Sciences, Edith Cowan University, Joondalup, Perth WA 6027, Australia; r.sambell@ecu.edu.au (R.S.); amanda.douglas-watson@hotmail.com (A.D.-W.); a.devine@ecu.edu.au (A.D.)

**Keywords:** charitable food sector, food insecurity, food literacy, nutrition education, training, tertiary education

## Abstract

Food literacy is seen as a key component in improving the increasing levels of food insecurity. While responsibility for providing training falls on the charitable service organizations, they may not have the capacity to adequately reach those in need. This paper proposes a tertiary education - (university or higher education) led model to support the food literacy training needs of the food charity sector. A cross-sectional study comprised of online surveys and discussions investigated food services offered by Western Australia (WA) and Australian Capital Territory (ACT) agencies, food literacy training needs for staff, volunteers and clients, and challenges to delivering food literacy training programs. Purposive sampling was used, and ACT and WA charitable service originations (survey: ACT *n* = 23, WA *n* = 32; interviews: ACT *n* = 3, WA *n* = 2) were invited to participate. Findings suggest organizations had limited financial and human resources to address the gap in food literacy training. Nutrition, food budgeting, and food safety education was delivered to paid staff only with limited capacity for knowledge transfer to clients. The Food Literacy Action Logic Model, underpinned by a tertiary education engagement strategy, is proposed to support and build capacity for organizations to address training gaps and extend the reach of food literacy to this under-resourced sector.

## 1. Introduction

Australia is ranked as the 15th most food-secure country in the world [1]. The prevalence of food insecurity amongst the Australian population is estimated to be 4% [2]. Within Australia, the most vulnerable to food insecurity include: Aboriginal and Torres Strait Islander People; people experiencing homelessness, culturally and linguistically diverse groups; elderly; disabled people; young people; and low-income earners [3,4,5]. The prevalence of food insecurity has been reported to be as high as 71% among newly arrived refugees [2,3,6], and national data suggests one in five Aboriginal and Torres Strait Islander households ran out of food and had not been able to afford to buy more in the previous 12 months [7]. Food insecurity in youth has been associated with poor mental health, particularly hyperactivity/inattention, and can manifest into depression and suicide [8,9] and, in adults, has been is associated with poorer self-reported health status [10].

Food insecurity is defined as the “*limited or uncertain availability of nutritionally adequate and safe foods or limited or uncertain ability to acquire acceptable foods in socially acceptable ways*” [11]. The food insecurity spectrum ranges from being mildly food insecure, whereby there are challenges to accessing adequate food or being able to cook food, to experiencing hunger and malnutrition [12]. A primary reason for food insecurity is poverty [13], however, other environmental factors including organizational, community, and government structures may impede food security [14]. The resultant increase in individual risk against the four pillars of food security, food access, availability, utilization, and stability [14,15], is seen especially in vulnerable groups. Furthermore, there is much evidence in relation to the link between food insecure individuals and chronic disease [16,17,18,19].

Improving an individuals’ food literacy has been identified as one strategy to improve food insecurity [15,16,20,21,22] and build resilience to cope with being food insecure [23]. Food literacy is defined as the relative ability to understand the nature of food, its importance, and understand how to use information about food for better health outcomes [24]. The key aspects of food literacy include food access, planning and management, selection, cooking, eating, and nutrition [24]. Research suggests that poor food literacy is a barrier for nutritious food access, wherein individuals with limited nutrition knowledge and skills are more likely to purchase ultra-processed foods [24,25], and have reduced individual creativity in meal preparation and awareness of health benefits of food [26]. An Australian food charity organization [27] reported that food literacy interventions have positively impacted those from low socio-economic demographics, with participation in multiple nutrition education interventions identified as a key component to reducing food insecurity [15].

Community service organizations are often the first point of call for those seeking assistance, and through their many programs, provide access to food and food literacy programs [28]. Despite the availability of food literacy courses and resources for organizations’ staff, volunteers, and clients in Australia [29,30,31], not all of these groups receive training, thus limiting knowledge transfer of food literacy. From a knowledge brokerage perspective, food literacy programs delivered by a familiar community service agency has the potential to provide a safe learning environment; and previous research has demonstrated improved nutrition skills, food choices, preparation, and cooking skills thus reducing the risk of chronic diseases [26]. Therefore the aim of this study was to identify the food services offered by agencies in Western Australia (WA) and Australian Capital Territory (ACT) regions; scope the needs of food literacy training for staff, volunteers, and clients from a staff perspective; investigate challenges of food rescue organizations engagement with existing food literacy training programs; and propose a tertiary education (university or higher education) led model to support the food literacy training needs of the food charity sector.

## 2. Materials and Methods

### 2.1. Study Design

A cross-sectional design comprising an online survey to community service agencies and discussions with Chief Executive Officers or Managers from five key food rescue organizations (WA (*n* = 2) and ACT (*n* = 3)) was used for the study. Community service agencies that provide programs within the Perth metropolitan area of WA and within Canberra, ACT, were surveyed to scope the current delivery of food literacy programs and training for staff and volunteers and the needs of clients from the staff perspective. Organizations in WA and ACT were randomly selected, based on organization type (emergency relief provider, food rescue organization, charitable food pantry, and providers of services to those who are homeless), were invited to participate in the discussion. The purpose of the discussions was to (1) expand the survey findings; (2) provide differences in context between jurisdictions; and (3) determine challenges to food literacy training and delivery from the organizational perspective. The concept of food literacy was outlined by the interviewer to the interviewees of the food rescue organizations. As discussions were informal and explorative in nature, the conversation flowed freely around the topic area of food literacy, which encompassed nutrition literacy. The Edith Cowan University Human Research Ethics Committee (12509) approved the study, and a reciprocal agreement was provided by the University of Canberra Human Research Ethics Committee. Informed consent was obtained from participants prior to completing the survey and discussions.

### 2.2. Recruitment of Agencies

Purposive sampling was undertaken to conduct this study. Community service organizations that provided community food programs were identified using publicly identifiable information (WA *n* = 112; ACT *n* = 56). Where an email address was publicly available, an initial email invitation to participate and an online survey link was sent (*n* = 99, WA; *n* = 50, ACT). Agencies without this information were contacted by telephone and asked to participate and provided the link or completed the survey with the contact over the phone (*n* = 13, WA; *n* = 6, ACT). To improve survey completion, organizations were reconducted at fortnightly intervals between November 2015–February 2016 (WA) and May–July 2016 (ACT).

### 2.3. Survey Development

An investigator-generated 23 item survey was designed to scope the range of primary services provided, demographics of clients, types of food and food services offered, use of food literacy programs, food literacy training needs of staff, volunteers, and needs of clients from the staff perspective. All closed items had nominal or ordinal response categories. The survey was developed by the researchers from (Removed for blinding). Content and face validity of the survey was conducted prior to administration in this study by key experts from three independent WA food rescue organizations and community service agencies. The final survey was administered online through Qualtrics LLC, Provo, Utah [32], a copy of which can be obtained from the authors.

### 2.4. Data Analysis

Quantitative data were analyzed using SPSS, Version 21 (IBM Corporation, Chicago, IL, USA). Basic descriptive and frequency analyses were conducted. Transcripts were coded using the thematic analysis process, as outlined by Braun and Clarke [33]. Initial coding of the data identified a consistent alignment of codes across the transcripts with the four pillars of food security: Availability, access, utilization, and stability [14]. These themes also aligned with the aims of the project, in particular aim 2, and informed the development of the model, aim 3. Codes that did not align with the four pillars were not identified during the coding. The coding process was conducted by author R.S. and reviewed by authors T.L. and A.D.

## 3. Results

### 3.1. Respondent Demographics

Community service organization Chief Executive Officers or Managers (*n* = 55) completed the online survey with a response rate of 29% (*n* = 32) for WA and 41% (*n* = 23) for ACT. Organizations provided more than one primary service to a range of client groups (Table 1), however, in both ACT and WA, the most reported primary service was, “welfare/homelessness service” (ACT 43% *n* = 10; WA 59% *n* = 19). There was greater variation between the proportion and types of client groups serviced by the organizations between ACT and WA, such that the primary client group in the ACT included “low income” (*n* = 20, 87%) and “Aboriginal and/or Torres Strait Islander people” (*n* = 14, 61%), whereas, both of these groups were equally reported as the main client group in WA (*n* = 26, 81%). The “homeless” (*n* = 23, 72%) and “asylum seekers, migrants or refugees” (*n* = 19, 59%) groups were also reported as key recipients in WA.

Forty-two percent of organizations offered case management services to their clients (ACT 30% *n* = 7; WA 50% *n* = 16). Of these, 87% (*n* = 20/23) included a food literacy program within the case management structure. Programs were either conducted on one occasion (Total *n* = 5; WA *n* = 5) or once a week for several weeks (Total *n* = 15; ACT *n* = 7; WA *n* = 8). Despite 58% of organizations not providing case management and nutrition services to their clients, 60% of total survey respondents (*n* = 33/55) indicated they had access to an onsite kitchen: 18% had access to a domestic size kitchen (Total *n* = 10; 13% ACT *n* = 3; WA 22% *n* = 7) and 42% had access to a commercial kitchen (Total *n* = 23; ACT 52% *n* = 12; WA 34% *n* = 11).

The community service organizations in ACT and WA provided a variety of food services and foods (Total *n* = 19; ACT *n* = 9; WA *n* = 10) to approximately 2500 clients per week (ACT *n* = 1100; WA *n* = 1400, Table 1). The primary food services offered included prepared food (Total *n* = 41; ACT *n* = 15; WA *n* = 19), emergency relief parcels (Total *n* = 31; ACT *n* = 11; WA *n* = 20) and food pantries (Total *n* = 19; ACT *n* = 9; WA *n* = 10). Food provision included fruit (Total *n* = 23; ACT *n* = 11; WA *n* = 12), hot and cold beverages (Total *n* = 20; ACT *n* = 10; WA *n* = 10), snacks (Total *n* = 16; ACT *n* = 9; WA *n* = 7), sandwiches (Total *n* = 15; ACT *n* = 10; WA *n* = 5) and hot meals (Total *n* = 15; ACT *n* = 10; WA *n* = 5).

### 3.2. Food Literacy Training

The scope of the food literacy training programs listed in the survey were food safety and handling, food budgeting, nutrition and cooking. Food safety and handling was the primary training delivered to paid staff and a priority area in both ACT and WA for volunteers. Similarly, centers in ACT (*n* = 7/23) and WA (*n* = 8/32) organizations, provided some training in food budgeting, nutrition, and cooking for clients. Face-to-face or online training was delivered by a variety of educational providers or provided from within the organization.

Organizations budgeted between $ 0 and $ 5000 per annum (pa) for food literacy training for staff; on average, ACT and WA reported $ 1100 pa and $ 400 pa respectively. The budget reduced considerably for ACT and WA volunteers ($ 400 pa vs. $ 100 pa respectively) and clients ($ 36 pa vs. $ 20 pa, respectively). Reflective of the reported budgets, 32–38% of organizations did not provide any food literacy training for paid staff, and 50–65% did not provide training for volunteers and clients.

### 3.3. Main Challenges in Conducting Food Literacy Programs

The primary challenges experienced by the community service organizations, from the staff perspective when running food literacy programs for clients were similar between ACT and WA. This included inadequacies in client’s nutrition knowledge (ACT *n* = 10 (40%); WA *n* = 15 (60%)), motivation to prepare healthy food (ACT *n* = 9 (39%); WA *n* = 14 (61%)); and skills (ACT *n* = 9 (50%); WA *n* = 9 (50%)). Organizational challenges in WA included lack of access to food for clients (*n* = 9), time (*n* = 9) and insufficient funding to supplement the food supplied for programs (*n* = 9). ACT lacked kitchen access and equipment (*n* = 6).

With unlimited time, facilities and budget, the three priority areas for training staff and volunteers were similar between ACT and WA. For staff, basic nutrition education was prioritized in both jurisdictions; WA prioritized food budgeting (*n* = 14); ACT prioritized food safety (*n* = 10). Priorities for WA and ACT volunteers were nutrition education (*n* = 12) and food safety (*n* = 12) and for clients, basic nutrition education (*n* = 16) and food budgeting (*n* = 16) respectively.

### 3.4. Key Organisation Views

Discussions with Chief Executive Officers or Managers from five key organizations supplemented the survey data and highlighted the role that organizations, agencies, and institutions including universities can play to improve food literacy and reduce food insecurity. Transcripts were coded against the four pillars of food security [14]: Availability, access, utilization and stability. The potential roles are outlined in relation to these pillars below.

#### 3.4.1. Availability

Organizations reported relying on food donations to provide fruit and vegetables and other foods for use in food literacy programs. Program sustainability was affected by the amount of food required beyond donations. Organizations indicated they would benefit from collaborative funding opportunities, thus providing consistent food availability at their services.

#### 3.4.2. Access

Adequate access is fundamental to an individual’s food security status. The organizations’ ability to support knowledge transfer around access through food literacy programs was proposed to support food security. The programs needed to include the who–who delivers the program; the level–appropriate program tailoring for clients; and, the where–resources to deliver program, location, and access to kitchen facilities, to ensure stability and client engagement. Further discussions highlighted that the delivery of the food literacy programs was critical and required expertise in food and nutrition, pedagogy, cooking skills, and target relevance:

“*From what we found that people are saying to us ‘we don’t want celebrity chefs teaching us how to cook’. It frightens those people that are vulnerable.*”

“*I would say that they (the person running the program) have to be confident at cooking. Again it is about being able to throw things in, being able to be a bit flamboyant with the way they are doing it. Other than just by the book. I think it has to have a bit of flexibility in it for it to suit.*”

It was suggested that without consideration of these factors, utilization of food literacy programs and food supplied would be reduced, thus lessening the client and organizational impact on food security.

“*When people are living in cars, how do they cook? The little stove, doing a bit of camp cooking.*”

“*That is the problem with the end user, the person getting the box of food. They are often people with very limited–they might be homeless or be in a hostel–ability to actually choose and prepare food for themselves. Their circumstances are that they get what they can and its often soup kitchen or prepared by a charity and given straight to them.*”

Similar to survey responses, human resources, staff and volunteer turnover, and limited space and facilities to conduct food literacy programs were identified as challenges. Funding in particular was found to be the biggest challenge to overcome.

“*Sustainable funding to keep the ball rolling. That’s our biggest issue and particularly in those high need areas where there is no money…for human resources to keep the program running. This is important to inspire and motivate.*”

#### 3.4.3. Utilization

Food literacy education provides an opportunity to upskill staff, volunteers, and clients to priorities nutrient-rich food consumption. The content and tools of food literacy programs should embed contextualized and well-evidenced information incorporating simple and robust recipes with common and inexpensive ingredients.

“*We think we have to measure everything, and I think that sometimes frightens people… When you take the measurement out it also takes the likelihood of it being wrong which can put people off. The fear of it not being as it should be … What I think you need to do is really pare it back. It has got to be simple. I cook like that I don’t measure anything. Teach people to do it by taste.*”

“*Another thing we found was that some of the barriers to producing this information were the client’s knowledge. Maybe some of the programs that are out there are too high a level for those clients.*”

#### 3.4.4. Stability

Food insecurity and subsequent nutrition insecurity can be outcomes of unstable food supply or lack of infrastructure to provide adequate food and/or training to empower individuals to overcome their food security status. The potential of training programs to build food literacy skills in clients could, in turn, go some way in offering a pathway to a more stable food supply. The organizations, however, had concerns given their limited capacity to provide food literacy programs themselves:

“*……What I think we need to be thinking about in this instance is how we cannot just roll this out in Perth metropolitan area, but HOW can we roll it out into the regional centers.*”

A tertiary education partnership model, which relies on student engagement, a flexible framework with relevant, authentic teaching, and learning structures were discussed with each of the key organizations to address the limited capacity and extend their reach of food literacy training. While the proposal of a partnership model was looked upon favorably, an organization stated that:

“*As long as you’ve got a framework (within this model)–this is your outcome, how you get there might be determined by your clients on the day.*”

All organizations agreed that a model requires buy-in from stakeholders within community service and food rescue organizations and potential partnerships with other commercial businesses or sectors outside the charitable food sector to be sustainable and successful:

“*It is an ownership as well. People like to be involved but they like to own it as well. They would own it by financially contributing.”*

## 4. Discussion

This study highlights the challenges experienced by the charitable food sector to improve food security through the provision of food literacy programs. Despite the varying geographical locations, similar findings across the jurisdictions (WA, ACT) were evident, including the fact that the primary services being offered were welfare/homeless services and for low income and Aboriginal and/or Torres Strait Islander peoples. While the amount of funding provided by ACT and WA organizations on food literacy training differed, limited financial support was evident, suggesting other options for food literacy training need to be considered. The priority focus for the training of staff, volunteers, and clients was food safety and handling. Nutrition and food budgeting were prioritized, but lack of funding and facilities limited actual delivery. External organizations provided food literacy training, resulting in additional costs for the organizations, thus limiting uptake and reach.

**Figure 1:**
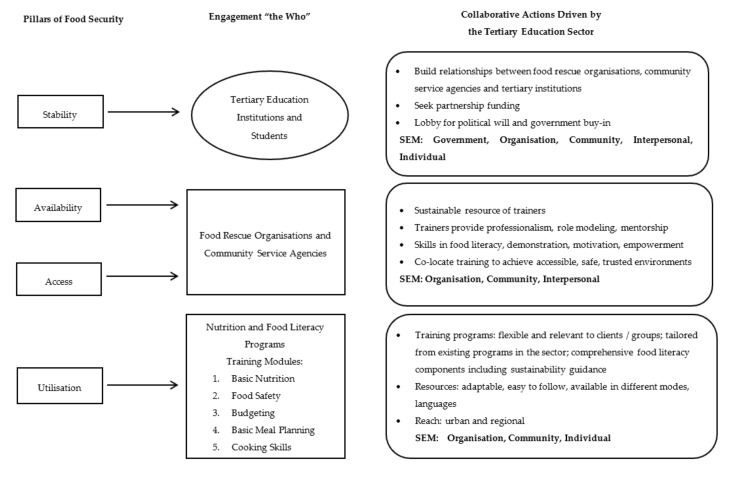
Food Action Logic Model: A schematic representation of collaborative actions driven by the tertiary education sector to address engagement with food rescue and community service organization’s to improve food security via a food literacy education model. SEM: Socio-ecological Model.

To provide sustainable programs, charitable food organizations acknowledged that partnerships with business, education, and health sectors were required. Engaging support was difficult due to changing external interests, poor funding models, and the absence of a logical framework to drive the process. Analysis of the survey findings and discussions supported the formulation of a Food Literacy Action Logic Model (Figure 1). This model outlines how the tertiary education sector can partner with the community service and food rescue organizations to provide sustainable food literacy training, foster skill development, and extend reach.

The Food Literacy Action Logic Model is premised upon the four pillars of food security: Availability, access, utilization, and stability [14,15]; and, incorporates the Socio-Ecological Model (SEM) of Health Promotion [34]. The SEM provides a framework to understand the complex and multifaceted interactions within the social system [34]. Accepted globally, the SEM is used to develop and evaluate nutrition, social and health programs [35] and analyze the importance of health/disease related decisions [36]. The SEM has five hierarchical levels: Individual, Interpersonal, Organizational, Community, and Policy/Enabling environments [34]. To achieve food security for the individual, community, and population, it is essential that all four pillars are supported and encompass all SEM levels.

The Food Literacy Action Logic Model guides the tertiary education sector in supporting all four pillars of food security. Engagement with the community services sector supports relationships between stakeholders at all levels of the SEM framework [34]. For example, “Policy/Enabling environments” includes partnerships with national, state, territory and local governments and food industry to improve funding and resources to ensure sustainable food supply and programs. Partnerships between food rescue organizations, food and non-food businesses, and the tertiary sector are needed to increase funding opportunities to improve the sustainability of food literacy programs. In turn, partnerships at the community and organization levels foster trusting environments, thus improving client’s access to food and knowledgeable trainers are likely to reduce attrition from programs [37]. The pillar of utilization of food is enhanced by a multi-strategy approach to food literacy education, as demonstrated by the Food Literacy Action Logic Model [37].

### Limitations

Despite a low response rate, the similarity in responses within each jurisdiction may imply a representative sample albeit from purposive sampling from a comprehensive publicly available database. Survey responses, including the training needs of clients, were based upon the staff perspective only. Discussions with the sector were informal and explorative in nature to determine the needs of the sector to build models for support.

## 5. Conclusions

Food literacy is one of the many strategies used to address food insecurity. A sustainable model showcasing collaboration with the tertiary education sector and the charitable food sector has been proposed to engage a sustainable student workforce to deliver food literacy training to staff and volunteers in order to improve knowledge transfer to clients. The delivery of flexible, relevant, and authentic food literacy programs tailored for regional and urban environments and contextualized for the client groups will contribute to greater food utilization and social skills for the client with an aim to improve short and long-term health.

## Figures and Tables

**Table 1:** Demographic comparison between Western Australia (WA) and Australian Capital Territory (ACT) charitable organizations.

Category	ACT (*n*)	WA (*n*)	Total (*n*)
	23	32	55
Number of Paid Staff (approx.)	55	117	172
Number of People Volunteering (approx.)	166	628	894
Clients Attending and Receiving Food (approx.)	1100	1400	2500
Primary Services Provided by Organisations Disability Services Welfare/Homelessness Services Health Organisation Sport/Social and Community Programs Food Program Other programs not listed Mental Health Alcohol, Drugs and Rehabilitation Domestic Violence	0 10 1 1 7 4 2 1 0	1 19 1 4 6 2 0 0 1	1 29 2 5 13 6 2 1 1
Client groups Aboriginal and Torres Strait Islander People Asylum Seekers, Migrants or Refugees Homeless Low Income Clients Other than those listed Individuals with a Disability Individuals with Mental Health Conditions Individuals with Alcohol and Drug Problems Family Specific Focus	14 6 12 20 10 1 2 2 4	26 19 23 26 5 1 0 0 2	40 25 35 46 15 2 2 2 6
